# Symptom-based clusters in people with ME/CFS: an illustration of clinical variety in a cross-sectional cohort

**DOI:** 10.1186/s12967-023-03946-6

**Published:** 2023-02-10

**Authors:** Anouk W. Vaes, Maarten Van Herck, Qichen Deng, Jeannet M. Delbressine, Leonard A. Jason, Martijn A. Spruit

**Affiliations:** 1 Department of Research and Development, Ciro, Horn, The Netherlands; 2 Department of Respiratory Medicine, Nutrim School of Nutrition and Translational Research in Metabolism, Faculty of Health, Medicine and Life Sciences, Maastricht University, Maastricht, The Netherlands; 3 Faculty of Rehabilitation Sciences, REVAL Rehabilitation Research Center, BIOMED Research Institute, Hasselt University, Diepenbeek, Belgium; 4 Center for Community Research, DePaul University, Chicago, USA

**Keywords:** Myalgic encephalomyelitis, Chronic fatigue syndrome, ME/CFS, Symptoms, Clusters

## Abstract

**Background:**

Myalgic encephalomyelitis (ME)/chronic fatigue syndrome (CFS) is a complex, heterogenous disease. It has been suggested that subgroups of people with ME/CFS exist, displaying a specific cluster of symptoms. Investigating symptom-based clusters may provide a better understanding of ME/CFS. Therefore, this study aimed to identify clusters in people with ME/CFS based on the frequency and severity of symptoms.

**Methods:**

Members of the Dutch ME/CFS Foundation completed an online version of the DePaul Symptom Questionnaire version 2. Self-organizing maps (SOM) were used to generate symptom-based clusters using severity and frequency scores of the 79 measured symptoms. An extra dataset (n = 252) was used to assess the reproducibility of the symptom-based clusters.

**Results:**

Data of 337 participants were analyzed (82% female; median (IQR) age: 55 (44–63) years). 45 clusters were identified, of which 13 clusters included ≥ 10 patients. Fatigue and PEM were reported across all of the symptom-based clusters, but the clusters were defined by a distinct pattern of symptom severity and frequency, as well as differences in clinical characteristics. 11% of the patients could not be classified into one of the 13 largest clusters. Applying the trained SOM to validation sample, resulted in a similar symptom pattern compared the Dutch dataset.

**Conclusion:**

This study demonstrated that in ME/CFS there are subgroups of patients displaying a similar pattern of symptoms. These symptom-based clusters were confirmed in an independent ME/CFS sample. Classification of ME/CFS patients according to severity and symptom patterns might be useful to develop tailored treatment options.

**Supplementary Information:**

The online version contains supplementary material available at 10.1186/s12967-023-03946-6.

## Introduction

Myalgic encephalomyelitis (ME)/chronic fatigue syndrome (CFS) is a serious long-term, multi-system disease, which is often characterized by debilitating fatigue that lasts at least 6 months and cannot be explained by other underlying medical conditions. Globally, about 20 million people are thought to have ME/CFS [1].

The excessive fatigue is often accompanied by a variety of other symptoms, including post-exertional malaise (PEM), sleep problems, pain and cognitive problems [2]. These symptoms seriously affect daily life of people with ME/CFS, as they limit normal daily activities, social routines, work and/or leisure activities [3, 4]. As a result, people with ME/CFS often experience a reduced quality of life [5, 6].

As there is no diagnostic biomarker for ME/CFS, the diagnosis of the disease relies on self-reported symptoms [1]. To date, over twenty case definitions exist, each capturing a different subset of individuals based on their reported symptoms and functioning [2]. The Fukuda CFS Criteria [7], the Canadian ME/CFS Criteria (CCC) [8], the ME International Consensus Criteria (ME-ICC) [9], and the Institute of Medicine Criteria (IOM) [10] are the most commonly used. The lack of a clear case definition emphasizes the heterogeneity of the disease.

Preliminary evidence suggest that subgroups of people with ME/CFS exist, displaying a specific set or cluster of symptoms (i.e. a group of two or more symptoms that occur concurrently and are interrelated) which are relatively independent of other clusters [11]. However, clustering was based on a limited number of symptoms or non-core (i.e. less frequently observed) symptoms [12–17], and/or a non-validated questionnaires was used to assess symptoms [16, 18].

Investigating symptom-based clusters may provide a better understanding of the symptom experience of people with ME/CFS. This may contribute to a better understanding of the clinical complexity of ME/CFS, and in turn, may contribute to the development of more tailored symptom management strategies. Therefore, this study aimed to identify clusters in people with ME/CFS based on the frequency and severity of symptoms. In addition, an independent ME/CFS dataset was used to validate the symptom-based clustering.

## Methods

### Study design and participants

In this cross-sectional study, members of the Dutch ME/CFS Foundation (ME/CVS Stichting; https://mecvs.nl/) were invited to complete a web-based survey between January 26 and February 28 2022. Individuals who did not report being diagnosed with ME/CFS were excluded from the analyses.

Ethical approval for this study was waived by the medical ethics committee of Maastricht University because the Medical Research Involving Human Subjects Act (WMO) did not apply to this study (METC 2021-2797). Digital informed consent was obtained from all respondents at the start of the survey. Without providing informed consent, participants were unable to start the questionnaire.

### Measures

Participants completed an online version of the DePaul Symptom Questionnaire version 2 (DSQ-2), which is a self-report measure of ME and CFS symptomatology, demographics, and medical, occupational and social history [19]. The DSQ-2 has demonstrated to have a strong reliability and validity [19, 20] and is able to differentiate individuals with ME/CFS from healthy controls [21] and individuals with other chronic diseases [22, 23]. Furthermore, the DSQ-2 can be used to determine whether individuals meet the criteria for the Fukuda, CCC, ME-ICC and/or IOM case definition [20]. The questionnaire was translated into Dutch using a forward–backward translation procedure.

Participants reported the frequency and severity of 79 symptoms related to the illness over the past 6 months. Frequency of symptoms was rated on a 5-point Likert scale: 0 = none of the time, 1 = a little of the time, 2 = about half the time, 3 = most of the time, and 4 = all of the time. Similarly, severity of symptoms was rated on a 5-point Likert scale: 0 = symptom not present, 1 = mild, 2 = moderate, 3 = severe, and 4 = very severe.

Symptom scores were analyzed in two ways: (1) a composite variable was created by averaging the frequency and severity scores of each symptom and multiplying it by 25; the composite score of each symptom ranged from 0 to 100 points. A higher score indicated a higher symptom burden [20]; and (2) a binary “2/2 threshold” variable was created by examining the frequency and severity scores of each symptom; participants who reported ratings of two or higher for both frequency (i.e. about half the time, most of the time, or all of the time) and severity (i.e. moderate, severe, or very severe) were considered to have the symptom [20]. For all other scores the symptom was not present.

### Validation dataset

An extra dataset of 252 people with a self-reported diagnosis of ME/CFS from the United States (US) was used to assess the reproducibility of the symptom-based clusters [24]. Participants for the US database were recruited from email requests to national foundations, posts to online support groups, research forums and social media platforms. All participants provided digital informed consent and subsequently completed an online version of the DSQ-2. Part of these DSQ-2 data were published before [24].

### Statistics

Data are presented as median and interquartile ranges (IQR) for continuous data and as frequencies and proportions for categorical data. Moreover, a self-organizing map (SOM) was used to visualize the clustering of the patients. The SOM method can be viewed as a non-parametric regression technique that converts multi-dimensional data spaces into lower dimensional abstractions. A SOM generates a non-linear representation of the data distribution and allows the user to identify homogenous data groups visually.

Severity and frequency scores of the 79 measured symptoms were used. Therefore each participant had 158 features. Clustering was performed on MATLAB (R2022a, MathWorks, MA, USA), following its default SOM setting, except for the number of iterations for training the SOM, which was changed to 1000 [25]. The default random number generation of MATLAB was used to initialize all competitive units of the SOM, meaning that with the same input and SOM settings, the results are always the same. Further details on the clustering method are provided in Additional file 1.

A Kruskal–Wallis test, adjusted for multiple comparisons, was used to test differences in participant characteristics between clusters. Statistical analyses were conducted using SPSS 25.0 (IBM Corporation, NY, USA). A priori, the level of significance was set at p < 0.05. The Venn diagrams were generated with Meta-Chart (https://www.meta-chart.com/venn#/display) and intersection plots were made using the R package ‘UpSetR’ [26]. Finally, the trained SOM was applied to the data from the validation sample.

## Results

### Participant characteristics

**Fig. 1:**
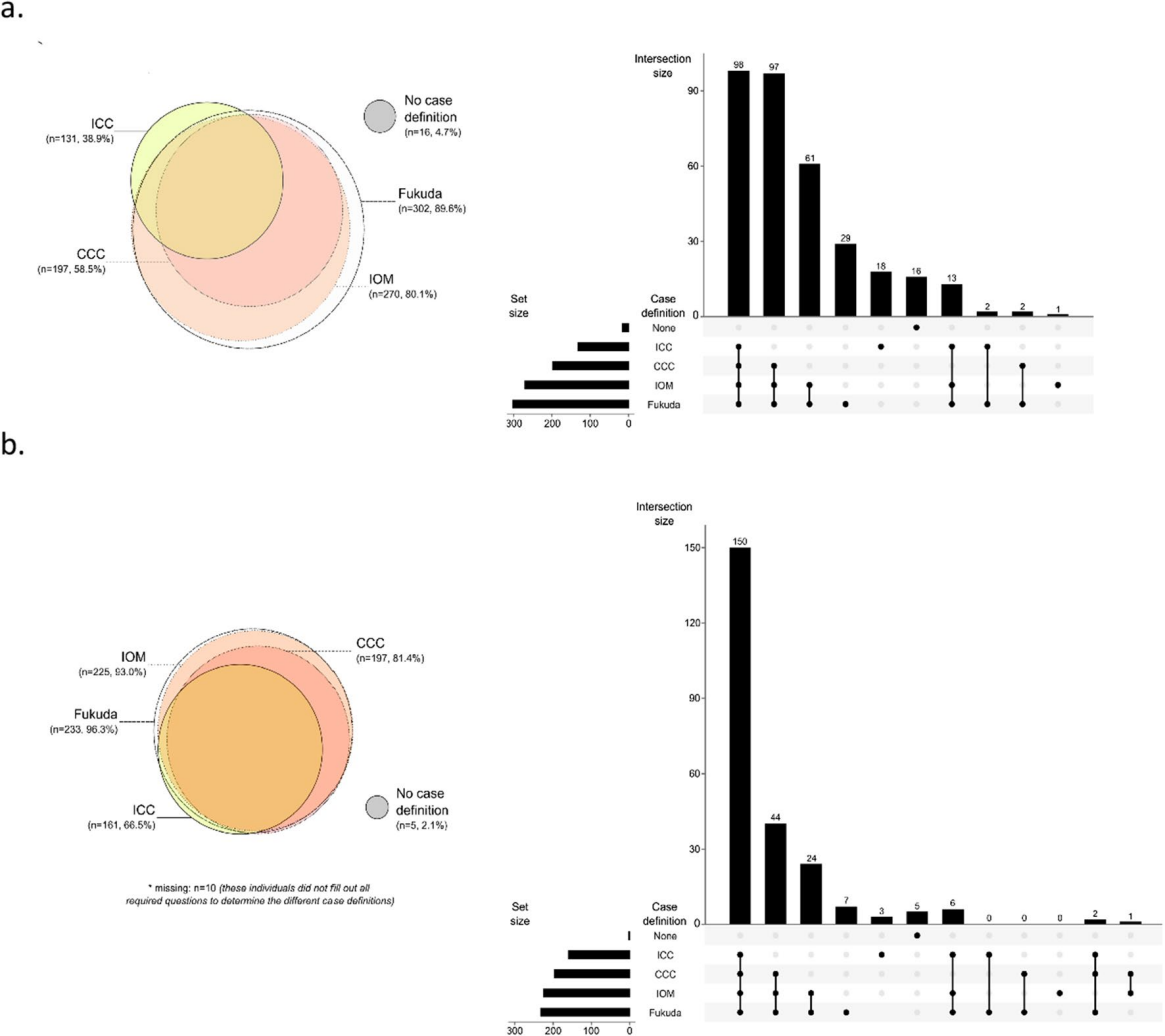
Proportional Venn diagrams and UpSet plots showing the overlap of different ME/CFS case definitions in the Dutch (**a**) and US (**b**) ME/CFS population. The different case definitions are displayed as horizontal bars on the lower left corner of the UpSet plot. Paired intersections are displayed as black dots; gray dots indicate the case definitions that are not part of the intersection. Black lines connecting 2 or more black dots indicate which case definitions form the intersections. The heights of the vertical bars indicate the intersection size (number of number of patients with indicated case definitions)

The link to the online questionnaire was send to 1392 members of the Dutch ME/CFS Foundation, of which 367 completed the questionnaire (response rate: 26%). Data from thirty participants were excluded, as they stated to not have been diagnosed with ME/CFS. So, data from 337 participants were used for the analyses (Table 1). In general, participants were mostly middle-aged women [82% female; median (IQR) age: 55 (44–63)] with a normal body mass index [24 (21–28) kg/m^2^]. Fifty-five percent of the participants were married or living with a partner. The majority of the participants had a medium or high education level and about two-thirds of them were incapacitated for work. Almost 90% of the participants fulfilled the Fukuda case definition, compared to 80%, 59% and 39% fulfilling the IOM, CCC and ME-ICC case definitions, respectively. More than a quarter of the participants met the criteria for all four different case definitions, whilst 5% of the participants met none of the abovementioned case definitions (Table 1, Fig. 1a).

**Table 1:** Participant characteristics

	Dutch database (n = 337)	US database (n = 252)	P-value
Sex, n (%)			0.242
Male	59 (17.5)	31 (12.3)	
Female	276 (81.9)	215 (85.3)	
Other/I don’t want to say	2 (0.6)	6 (2.4)	
Age, years	55 (44–63)	50 (38–58)^#^	< 0.001
BMI, kg/m^2^	23.9 (21.1–27.8)	–	
Ethnicity, n (%)			0.579
Black, African-American	1 (0.3)	–	
White	327 (97.0)	245 (97.2)	
American Indian or Alaska Native	–	1 (0.4)	
Asian or Pacific Islander	2 (0.6)	3 (1.2)	
Other	7 (2.1)	3 (1.2)	
Marital status, n (%)		^#^	< 0.001
Married or living with partner	185 (54.9)	121 (48.0)	
Living alone	127 (37.7)	77 (30.6)	
Widow(er)/divorced	25 (7.4)	54 (21.4)	
Children, n (%)			0.770
Yes	159 (47.2)	121 (48.0)	
Education level, n (%)^a^		^#^	0.001
Low	10 (3.0)	10 (4.0)	
Medium	127 (37.7)	62 (24.6)	
High	195 (57.9)	178 (70.6)	
Other/I don’t want to say	5 (1.5)	2 (0.8)	
Current work status, n (%)^b^		^#^	< 0.001
On disability	215 (63.8)	118 (46.8)	
Student	6 (1.8)	15 (6.0)	
Homemaker	26 (7.7)	13 (5.2)	
Retired	58 (17.2)	34 (13.5)	
Unemployed	23 (6.8)	35 (13.9)	
Working part-time	57 (16.9)	39 (15.5)	
Working full-time	9 (2.7)	20 (7.9)	
Case definition, n (%)^c^		^#^	< 0.001
Fukuda criteria	302 (89.6)	234 (92.9)	
Canadian clinical ME/CFS criteria	197 (58.5)	198 (78.6)	
International consensus criteria	131 (38.9)	162 (64.3)	
Institute of medicine	270 (80.1)	226 (89.7)	
Number of case definition, n (%)^c^		^#^	< 0.001
0	16 (4.7)	5 (2.1)	
1	48 (14.2)	10 (4.1)	
2	65 (19.3)	25 (10.3)	
3	110 (32.6)	52 (21.5)	
4	98 (29.1)	150 (62.0)	

^#^p < 0.05

^a^Classification according to the International Standard Classification of Education 2011[35]

^b^Multiple answers possible

^c^Missing values US database: n = 10 (participants did not fill out all the required questions to determine the different case definitions): Fukuda: n = 9; CCC: n = 9; IOM: n = 9; MEICC: n = 5; number of case definitions: n = 10

### Symptoms

The vast majority of the participants were experiencing fatigue (90.7% fulfilled the 2/2 threshold). Furthermore, PEM, sleep-related problems, neurocognitive problems and pain were frequently reported, whilst on average autonomic, neuroendocrine, and immune symptoms were less prevalent (Table 2). Participants reported a median (IQR) of 27 (19–37) symptoms (using the 2/2 threshold, Additional file 2).

**Table 2:** DSQ-2 symptoms

	Dutch database (n = 337)		US database (n = 252)	
	Composite score Mean (SD)	Participants at 2/2 threshold (%)	Composite score Mean (SD)	Participants at 2/2 threshold (%)
Fatigue/extreme tiredness	75.4 (17.5)	90.7	81.4 (15.1)	98.4
Post-exertional malaise				
Dead, heavy feeling after starting exercise	50.1 (26.9)	58.3	76.2 (24.7)	87.7
Next-day soreness or fatigue after everyday activities	66.6 (20.8)	80.7	75.3 (21.6)	90.9
Mentally tired after the slightest effort	61.7 (23.7)	72.8	68.2 (23.0)	86.1
Physically tired after minimum exercise	67.0 (21.7)	81.2	77.8 (20.6)	92.9
Physically drained or sick after mild activity	59.8 (22.8)	70.3	73.2 (22.5)	88.9
Muscle fatigue after mild physical activity	63.1 (25.7)	74.4	72.2 (26.1)	86.1
Worsening of symptoms after mild physical activity	67.7 (23.7)	78.5	78.8 (21.9)	92.9
Worsening of symptoms after mild mental activity	59.1 (25.9)	66.5	63.4 (27.0)	76.6
Difficulty reading (dyslexia) after mild physical or mental activity	41.2 (30.8)	40.6	44.6 (33.4)	49.2
Sleep				
Unrefreshing sleep	73.9 (22.3)	88.0	81.7 (19.1)	94.8
Need to nap daily	56.2 (32.3)	56.4	58.1 (30.0)	66.7
Problems falling asleep	47.4 (28.0)	49.9	55.9 (29.3)	63.9
Problems staying asleep	52.7 (28.6)	56.4	58.4 (30.5)	65.1
Waking up early in the morning (e.g. 3 AM)	43.2 (28.3)	43.1	48.9 (30.2)	49.6
Sleeping all day and staying awake all night	18.4 (22.8)	9.5	17.7 (27.2)	16.3
Daytime drowsiness	52.6 (26.9)	55.3	63.2 (26.9)	77.0
Pain				
Pain or aching in muscles	59.6 (24.8)	70.0	67.8 (26.0)	80.2
Joint pain	51.1 (29.9)	59.4	57.2 (32.9)	64.7
Eye pain	31.2 (24.6)	24.8	31.8 (28.8)	31.0
Chest pain	23.1 (21.8)	11.7	25.8 (25.7)	19.0
Bloating	38.2 (25.8)	37.6	47.1 (28.1)	50.4
Abdomen/stomach pain	36.4 (23.2)	30.8	42.1 (27.7)	43.7
Headaches	24.8 (23.8)	42.8	50.5 (26.3)	55.2
Aching of the eyes or behind the eyes	29.0 (24.6)	17.4	36.8 (29.8)	36.9
Sensitivity to pain	27.0 (30.0)	24.0	51.3 (32.9)	59.5
Myofascial pain	15.7 (25.7)	12.5	26.3 (34.0)	30.2
Neurocognitive				
Muscle twitches	26.9 (22.3)	16.6	34.4 (25.5)	29.8
Muscle weakness	44.7 (27.4)	45.2	63.5 (27.2)	74.6
Sensitivity to noise	54.7 (26.8)	57.8	61.0 (26.8)	69.8
Sensitivity to bright lights	47.3 (28.4)	46.0	53.2 (29.0)	55.6
Problems remembering things	52.2 (22.5)	58.0	67.9 (24.1)	84.5
Difficulty paying attention for a long period of time	60.8 (24.0)	73.3	70.4 (24.3)	86.1
Difficulty finding the right word to say, or expressing thoughts	48.5 (22.4)	50.7	61.1 (24.7)	75.4
Difficulty understanding things	33.3 (21.5)	23.2	48.5 (25.1)	55.2
Only able to focus on one thing at a time	57.1 (25.2)	67.6	64.9 (25.1)	78.6
Unable to focus vision	21.5 (23.6)	13.6	35.1 (27.4)	31.7
Unable to focus attention	34.6 (22.9)	27.2	50.9 (25.1)	63.1
Loss of depth perception	18.5 (25.6)	11.7	24.0 (29.5)	21.4
Slowness of thought	47.3 (26.4)	50.7	56.9 (25.5)	71.4
Absent-mindedness or forgetfulness	44.0 (22.6)	43.3	60.7 (24.5)	78.6
Feeling disoriented	20.2 (22.7)	11.2	35.9 (25.3)	29.4
Slowed speech	21.3 (22.7)	12.0	32.6 (27.1)	31.3
Poor coordination	30.0 (25.1)	21.3	46.2 (28.6)	52.8
Autonomic				
Bladder problems	22.1 (28.0)	18.3	35.6 (32.5)	34.5
Urinary urgency	34.5 (27.6)	31.3	39.7 (31.2)	42.9
Waking up at night to urinate	51.2 (29.2)	49.6	45.7 (32.0)	46.4
Irritable bowel problems	39.8 (29.7)	42.2	49.1 (32.7)	55.6
Nausea	28.4 (21.5)	16.6	33.8 (26.9)	30.6
Feeling unsteady on feet	34.2 (24.1)	23.7	44.0 (28.8)	46.4
Shortness of breath or trouble catching breath	27.9 (22.2)	17.7	39.8 (28.0)	37.7
Dizziness or fainting	31.3 (22.2)	19.6	42.1 (27.6)	43.7
Irregular heart beats	27.8 (23.0)	16.1	30.4 (26.8)	28.6
Heart beats quickly after standing	31.3 (28.9)	27.0	43.8 (32.6)	46.0
Blurred or tunnel vision after standing	21.9 (26.4)	16.6	30.5 (31.2)	29.0
Graying or blacking out after standing	32.5 (24.2)	23.2	22.7 (28.1)	17.5
Inability to tolerate an upright position	27.3 (27.9)	18.3	46.7 (34.3)	52.0
Neuroendocrine				
Losing weight without trying	7.1 (16.7)	4.1	13.2 (21.9)	7.5
Gaining weight without trying	18.3 (25.8)	13.9	36.3 (36.6)	41.3
Lack of appetite	19.3 (21.4)	11.4	30.3 (25.8)	25.4
Sweating hands	12.9 (21.2)	8.2	16.6 (25.7)	13.1
Night sweats	33.0 (27.3)	29.2	36.1 (30.5)	31.3
Cold limbs	51.9 (26.4)	58.3	46.9 (29.9)	48.4
Feeling chills or shivers	31.7 (23.1)	21.5	34.0 (26.3)	32.9
Feeling hot or cold for no reason	42.4 (24.8)	38.7	51.7 (26.9)	61.1
Felling like you have a high temperature	29.6 (22.6)	19.3	33.0 (27.2)	29.4
Feeling like you have a low temperature	20.7 (25.5)	15.8	24.7 (26.7)	21.8
Alcohol intolerance	15.1 (30.8)	14.2	40.1 (36.9)	34.1
Intolerance to extremes of temperature	42.3 (35.0)	41.7	64.9 (31.1)	72.2
Fluctuations in temperature throughout the day	30.3 (28.5)	26.4	45.2 (31.8)	48.0
Immune				
Sore throat	30.1 (20.4)	17.4	37.3 (25.7)	32.9
Tender/sore lymph nodes	23.4 (22.9)	14.4	37.5 (30.5)	36.5
Fever	9.2 (16.9)	1.6	16.1 (22.1)	9.9
Flu-like symptoms	36.1 (23.7)	28.3	51.8 (27.3)	57.1
Sensitivity to smells, food, medications, or chemicals	25.6 (27.1)	19.1	45.0 (32.7)	46.8
Viral infections with prolonged recovery periods	16.9 (24.7)	9.5	35.0 (32.7)	32.5
Sinus infections	15.2 (24.0)	10.4	21.9 (26.2)	19.4
Others				
Sensitivity to mold	18.3 (28.9)	16.6	27.2 (36.3)	30.6
Sensitivity to vibration	22.9 (29.9)	18.3	30.6 (35.0)	32.9

### Symptom-based clusters

**Fig. 2:**
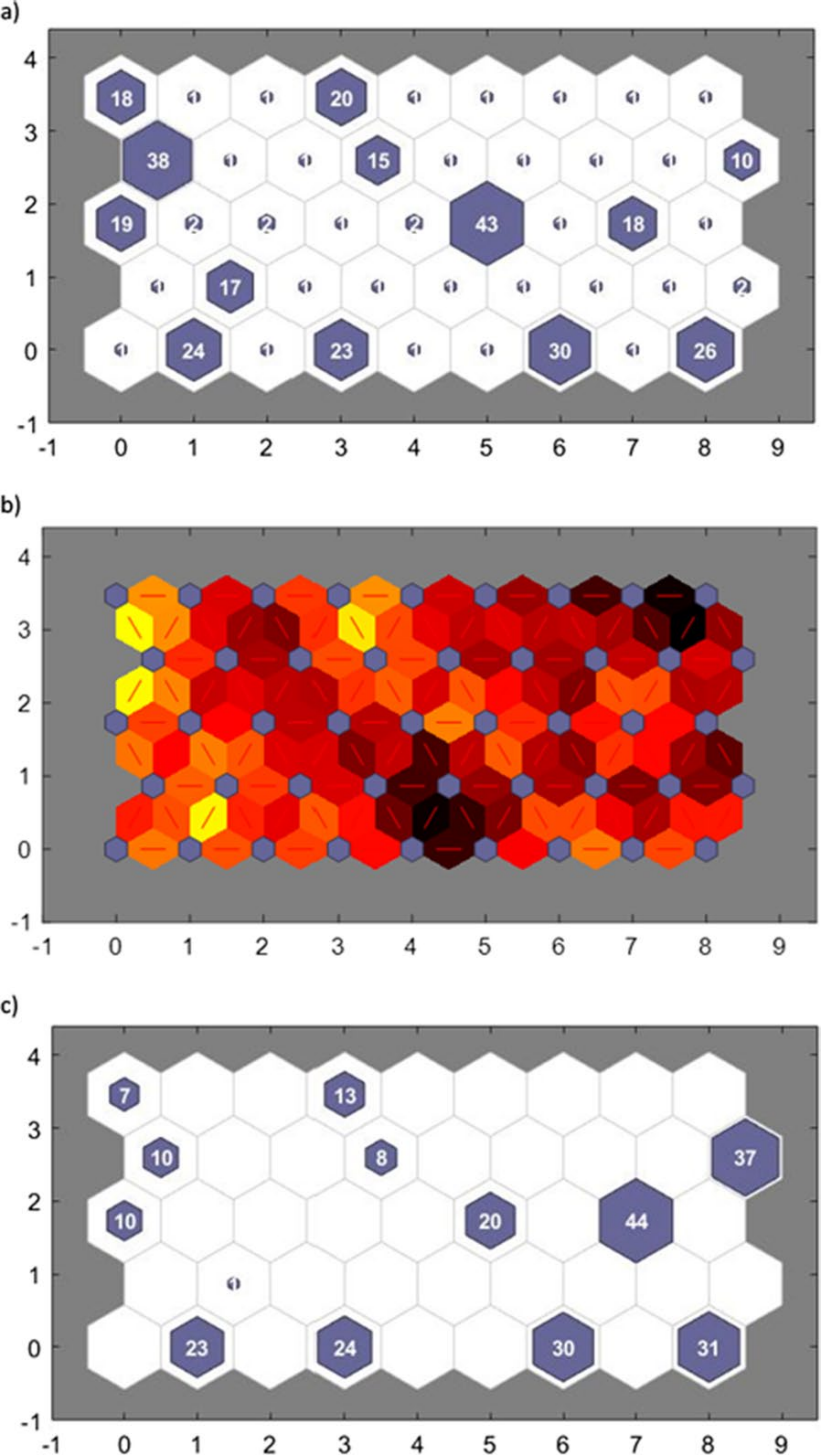
Symptom-based clusters using self-organizing maps. All clusters of patients are displayed in the direction of left to right and bottom to top. Each hexagon represents a cluster, and the number within a hexagon shows the number of patients in the cluster. The x-axis and y-axis indicate the number of clusters, starting from 0. In particular, coordinate (0,0) corresponds to Cluster 1, coordinate (1,0) corresponds to Cluster 2, etc. The 13 clusters including at least 10 patients are shown in purple. **a** Symptom-based clusters in Dutch ME/CFS population. **b** Connection between symptom-based clusters. The blue hexagons represent the clusters. The colors in the regions containing the red lines indicate the distances between clusters: darker colors represent larger distances (less similarity between the two clusters), lighter colors represent smaller distances (more similarity between the two clusters). **c** Symptom-based clusters in US population

Forty-five clusters were identified, of which 13 clusters included ≥ 10 patients (Fig. 2a, b). In general, key features of these 13 clusters can be summarized as follows:

- Participants in *Cluster 2* (n = 24) were characterized by low frequency and severity scores for problems related to dizziness/fainting, stomach ache and problems staying asleep.
- Participants in *Cluster 4* (n = 23) were characterized by low frequency and severity scores for urinary problems and higher frequency scores for increased heart rate by standing.
- Participants in *Cluster 7* (n = 30) were characterized by increased frequency scores for cognitive impairments.
- Participants in *Cluster 9* (n = 26) were characterized by high frequency and severity scores for dizziness/fainting, feeling unsteady on their feet and sensitivity/intolerance to smell and alcohol.
- Participants in *Cluster 11* (n = 17) were characterized by high frequency and severity scores for impaired day–night rhythm but low frequency and severity scores for muscle weakness and coordination problems.
- Participants in *Cluster 19* (n = 19) were characterized by low frequency scores for physical fatigue, symptoms after exercise and irritable bowel problems.
- Participants in *Cluster 24* (n = 43) were characterized by high frequency and severity scores for sensitivity to sound, sleeping problems and symptoms after exercise.
- Participants in *Cluster 26* (n = 18) were characterized by a relatively high symptom burden combined with high frequency and severity scores for temperature related symptoms and pressure pain.
- Participants in *Cluster 28* (n = 38) were characterized by a relatively low symptom burden and low frequency and severity scores for muscle related problems.
- Participants in *Cluster 31* (n = 15) were characterized by high frequency and severity scores for sensitivity/intolerance to mold and temperature and stomach/bowel problems.
- Participants in *Cluster 36* (n = 10) had the highest symptom burden (i.e. highest frequency and severity of symptoms).
- Participants in *Cluster 37* (n = 18) had the lowest symptom burden (i.e. lowest frequency and severity of symptoms).
- Participants in *Cluster 40* (n = 20) were characterized by low frequency and severity scores for symptoms after physical and mental exercise, muscle fatigue and problems with focus on one thing.

Participants in Clusters 28 and 37 reported a significantly lower number of symptoms compared to participants in Clusters 4, 7, 9, 24, 26, 31 and 36, whilst the number of symptoms in Clusters 36 was significantly higher compared to all other clusters, except for Clusters 7, 9 and 26 (p < 0.05; Table 3).

**Table 3:** Participant characteristics per cluster

	Cluster 2 (n = 24)	Cluster 4 (n = 23)	Cluster 7 (n = 30)	Cluster 9 (n = 26)	Cluster 11 (n = 17)	Cluster 19 (n = 19)	Cluster 24 (n = 43)	Cluster 26 (n = 18)	Cluster 28 (n = 38)	Cluster 31 (n = 15)	Cluster 36 (n = 10)	Cluster 37 (n = 18)	Cluster 40 (n = 20)
Sex, n (%)													
Male	6 (25.0)	2 (8.7)	8 (26.7)	4 (15.4)	6 (35.3)	8 (42.1)	4 (9.3)	3 (16.7)	2 (5.3)^1^	3 (20.0)	1 (10.0)	6 (33.3)	2 (10.0)
Female	18 (75.0)	21 (91.3)	22 (73.3)	22 (84.6)	11 (64.7)	10 (52.6)	39 (90.7)	15 (83.3)	36 (94.7)	12 (80.0)	9 (90.0)	12 (66.7)	17 (85.0)
Other/I don’t want to say	–	–	–	–	–	1 (5.3)	–	–	–	–	–	–	1 (5.0)
Age, years	52 (47–61)	38 (27–48)^2^	55 (45–58)	55 (39–60)	51 (39–62)	60 (50–70)	59 (51–64)	52 (44–61)	57 (39–63)	52 (43–59)	52 (41–58)	61 (53–71)	62 (52–70)
BMI, kg/m^2^	24.6 (21.4–29.0)	20.9 (20.3–27.6)	26.0 (21.7–30.7)	23.4 (21.9–26.5)	24.0 (19.6–26.2)	25.9 (21.5–31.2)	24.5 (22.3–28.6)	23.5 (21.2–26.1)	24.2 (21.4–29.6)	21.3 (20.3–25.3)	22.7 (21.0–27.9)	25.4 (22.8–26.8)	22.4 (19.8–28.1)
Ethnicity, n (%)													
Black, African-American	1 (4.2)	–	–	–	–	–	–	–	–	–	–	–	–
White	22 (91.7)	23 (100.0)	29 (96.7)	24 (92.3)	17 (100.0)	18 (94.7)	43 (100.0)	17 (94.4)	37 (97.4)	15 (100.0)	9 (90.0)	18 (100.0)	19 (95.0)
American Indian/Alaska Native	–	–	–	–	–	–	–	–	–	–	–	–	–
Asian or Pacific Islander	1 (4.2)	–	–	–	–	–	–	–	–	–	1 (10.0)	–	–
Other	–	–	1 (3.3)	2 (7.7)	–	1 (5.3)	–	1 (5.6)	1 (2.6)	–	–	–	1 (5.0)
Marital status, n (%)													
Married or living with partner	15 (62.5)	9 (39.1)	16 (53.3)	13 (50.0)	8 (47.1)	8 (42.1)	27 (62.8)	13 (72.2)	22 (57.9)	12 (80.0)	2 (20.0)	8 (44.4)	13 (65.0)
Living alone	8 (33.3)	12 (52.2)	10 (33.3)	11 (42.3)	8 (47.1)	7 (36.8)	14 (32.6)	5 (27.8)	15 (39.5)	3 (20.0)	6 (60.0)	8 (44.4)	6 (30.0)
Widow(er)/divorced	1 (4.2)	2 (8.7)	4 (13.3)	2 (7.7)	1 (5.9)	4 (21.1)	2 (4.7)	–	1 (2.6)	–	2 (20.0)	2 (11.1)	1 (5.0)
Children, n (%)													
No	12 (50.0)	16 (69.6)	16 (53.3)	15 (57.7)	9 (52.9)	9 (47.4)	24 (55.8)	9 (50.0)	22 (57.9)	8 (53.3)	4 (40.0)	7 (38.9)	9 (45.0)
Yes	12 (50.0)	7 (30.4)	14 (46.7)	11 (42.3)	8 (47.1)	10 (52.6)	19 (44.2)	9 (50.0)	16 (42.1)	7 (46.7)	6 (60.0)	11 (61.1)	11 (55.0)
Education level, n (%)^a^													
Low	1 (4.2)	–	–	–	–	2 (10.5)	1 (2.3)	3 (16.7)	1 (2.6)	–	1 (10.0)	1 (5.6)	–
Medium	7 (29.2)	8 (34.8)	13 (43.3)	9 (34.6)	6 (35.3)	10 (52.6)	19 (44.2)	6 (33.3)	10 (26.3)	7 (46.7)	5 (50.0)	1 (5.6)	7 (35.0)
High	16 (66.7)	14 (60.9)	17 (56.7)	16 (61.5)	11 (64.7)	7 (36.8)	23 (53.5)	9 (50.0)	27 (71.1)	8 (53.3)	4 (40.0)	15 (83.3)	13 (65.0)
Other/I don’t want to say	–	1 (4.3)	–	1 (3.8)	–	–	–	–	–	–	–	1 (5.6)	–
Current work status, n (%)^b^													
On disability	16 (66.7)	19 (82.6)	25 (83.3)	18 (69.2)	12 (70.6)	7 (36.8)	31 (72.1)	14 (77.8)	18 (47.4)	8 (53.3)	10 (100.0)	4 (22.2)^3^	10 (50.0)
Student	–	2 (8.7)	–	–	–	–	–	1 (5.6)	–	1 (6.7)	–	1 (5.6)	–
Homemaker	3 (12.5)	1 (4.3)	1 (3.3)	1 (3.8)	2 (11.8)	1 (5.3)	9 (20.9)	1 (5.6)	1 (2.6)	2 (13.3)	–	–	3 (15.0)
Retired	1 (4.2)	–	1 (3.3)	3 (11.5)	2 (11.8)	8 (42.1)^4^	7 (16.3)	2 (11.1)	6 (15.8)	3 (20.0)	1 (10.0)	8 (44.4)^4^	7 (35.0)
Unemployed	3 (12.5)	1 (4.3)	–	–	2 (11.8)	1 (5.3)	3 (7.0)	1 (5.6)	4 (10.5)	1 (6.7)	–	1 (5.6)	2 (10)
Working part-time	10 (41.7)^5^	2 (8.7)	–^5^	4 (15.4)	3 (17.6)	2 (10.5)	5 (11.6)	1 (5.6)	11 (28.9)	2 (13.3)	1 (10.0)	5 (27.8)	6 (30.0)
Working full-time	1 (4.2)	–	3 (10.0)	–	–	2 (10.5)	–	–	2 (5.3)	–	–	1 (5.6)	-
Case definition, n (%)													
Fukuda Criteria	20 (83.3)	21 (91.3)	26 (86.7)	26 (100.0)	17 (100.0)	16 (84.2)	39 (90.7)	15 (83.3)	34 (89.5)	14 (93.3)	5 (50.0)^6^	17 (94.4)	20 (100.0)
Canadian Clinical ME/CFS Criteria	12 (50.0)	15 (65.2)	26 (86.7)	26 (100.0)	9 (52.9)	3 (15.8)^7^	35 (81.4)	13 (72.2)	6 (15.8)^7^	11 (73.3)	5 (50.0)	–^7^	9 (45.0)
International Consensus Criteria	3 (12.5)	6 (26.1)	14 (46.7)	21 (80.8)^8^	6 (35.3)	3 (15.8)	19 (44.2)	18 (100.0)^9^	1 (2.6)	6 (40.0)	10 (100.0)^8^	–	8 (40.0)
Institute of Medicine	19 (79.2)	21 (91.3)	26 (86.7)	26 (100.0)	17 (100.0)	12 (63.2)	39 (90.7)	15 (83.3)	26 (68.4)	14 (93.3)	5 (50.0)	4 (22.2)^10^	16 (80.0)
Nr. of case definition, n (%)													
0	3 (12.5)	2 (8.7)	2 (6.7)	–	–	2 (10.5)	1 (2.3)	–	3 (7.9)	–	–	1 (5.6)	–
1	1 (4.2)	–	2 (6.7)	–	–	5 (26.3)	3 (7.0)	3 (16.7)	9 (23.7)	1 (6.7)	5 (50.0)	13 (72.2)^11^	2 (10.0)
2	9 (37.5)	2 (8.7)	–	–	6 (35.3)	7 (36.8)	4 (9.3)	–	20 (52.6)^12^	2 (13.3)	–	4 (22.2)	7 (35.0)
3	9 (37.5)	11 (47.8)	14 (46.7)	5 (19.2)	7 (41.2)	5 (26.3)	19 (44.2)	2 (11.1)	6 (15.8)	8 (53.3)	–^13^	–	7 (35.0)
4	2 (8.3)	5 (21.7)	12 (40.0)	21 (80.8)^14^	4 (23.5)	–	16 (37.2)	13 (72.2)^10^	–	4 (26.7)	5 (50.0)	–	4 (20.0)
Number or reported symptoms, median (IQR)^c^	22 (19–27)^15^	28 (23–31)^16^	38 (34–43)	41 (36–44)	23 (21–26)^15^	17 (14–22)^15^	31 (27–34)^16^	45 (41–53)	15 (11–19)^17^	28 (25–32)^16^	61 (59–62)	8 (4–11)^18^	21 (17–26)^15^

^1^p < 0.05 versus clusters 19; ^2^p < 0.05 versus clusters 7, 19, 24, 28, 37 and 40; ^3^p < 0.05 versus clusters 4, 7, 24, 26, and 36; ^4^p < 0.05 versus clusters 2, 4, and 7; ^5^p < 0.05 versus all other clusters; ^6^p < 0.05 versus clusters 4, 7, 9, 11, 24, 28, 31, 37, and 40; ^7^p < 0.05 versus clusters 4, 7, 9, 24, 26, and 31; ^8^p < 0.05 versus clusters 2, 4, 19, 28, and 37; ^9^p < 0.05 versus clusters 2, 4, 7, 11, 19, 24, 28, 31, 37, and 40; ^10^p < 0.05 versus clusters 2, 4, 7, 9, 11, 24, 26, 28, 31, and 40; ^11^p < 0.05 versus clusters 2, 4, 7, 9, 11, 19, 24, 26, 28, 31, and 40; ^12^p < 0.05 versus clusters 7, 9, 24, 26, and 36; ^13^p < 0.05 versus clusters 7 and 24; ^14^p < 0.05 versus clusters 2, 4, 19, 28, 37, and 40; ^15^p < 0.05 versus clusters 7, 9, 24, 26 and 36; ^16^p < 0.05 versus clusters 26 and 36; ^17^p < 0.05 versus clusters 4, 7, 9, 24, 26, 31 and 36; ^18^p < 0.05 versus clusters 2, 4, 7, 9, 11, 24, 26, 31, 36 and 40

^a^Classification according to the International Standard Classification of Education 2011[35]

^b^Multiple answers possible

^c^Using the 2/2 threshold for severity and frequency

Please see Additional file 3 for symptom scores of the different clusters. Please see Additional file 4 for the symptom scores of the remaining 42 clusters with < 10 patients.

### Clinical characteristics

Participant characteristics of the 13 largest symptom-based clusters are displayed in Table 3. On average, participants in cluster 4 were significantly younger compared to participants in clusters 7, 19, 24, 28, 37 and 40. Cluster 19 had a significantly higher proportion of males compared to Clusters 28. Prevalence of work disability was significantly lower in Cluster 37 compared to Clusters 4, 7, 24, 26 and 36. Please see Additional file 5 for the characteristics of the remaining 42 clusters with < 10 patients.

### Case definitions

Distribution across the different case definitions and number of case definitions that were met were significantly different between the 13 largest clusters. Generally, the proportion of patients fulfilling the different case definitions was highest in Cluster 9 and lowest in Cluster 19, 28 and 37. None of the participants in Cluster 37 fulfilled the CCC and ME-ICC case definition, and also the proportion of patients fulfilling the IOM case definition was lowest in Cluster 37. The proportion of patients fulfilling the Fukuda CFS Criteria was lowest in Cluster 36. In addition, Clusters 9, 26 and 36 had the highest proportion of participants fulfilling the ME-ICC case definition.

### Validation of symptom-based clusters

Participant characteristics of the US database are listed in Table 1. In general, these participants were slightly younger, were more often widower or divorced, had a higher education level and were less often on disability compared to the Dutch participants (all p < 0.05). The proportion of patients fulfilling the different case definitions and the total number of case definitions met was significantly higher in the US database compared to the Dutch database. Furthermore, US participants generally experienced a higher symptom burden compared to the Dutch participants (Table 2). Applying the trained SOM to the data of the validation sample, resulted in a similar symptom pattern compared the Dutch dataset (Fig. 2c).

## Discussion

This study demonstrated that people with self-reported ME/CFS can suffer from a variety of symptoms, besides severe fatigue. Indeed, these symptoms co-occurred in multiple specific patterns. Moreover, 5% of the people with ME/CFS did not meet the criteria of the most common case definitions. These findings were corroborated in an independent second sample of people with ME/CFS.

As expected, fatigue and PEM, which are considered key symptoms of ME/CFS, were reported across all of the symptom-based clusters. However, the clusters were defined by a distinct pattern of other symptoms. For example, *Cluster 7* was predominately characterized by cognitive symptoms, whilst *Cluster 26* was characterized by more temperature-related symptoms and pressure pain. Importantly, the different clusters are identified by a specific pattern of symptom severity and frequency, but this does not indicate that these symptoms are not present in other clusters.

Remarkably, 11% of the patients could not be classified into one of the 13 largest clusters (≥ 10 people), indicating that a considerable proportion of people with ME/CFS present an unique symptom pattern.

The current findings emphasize the large heterogeneity in symptoms in a sample of people with ME/CFS, and the complexity for clinicians to adequately monitor and treat these patients. Which symptom needs to be addressed first? Can different symptoms be addressed at the same time? Are symptoms responsive to pharmacological and/or non-pharmacological treatment?

Interestingly, when applying the identified clusters to the validation dataset, similar symptom patterns were found. This suggests that the symptom-based clusters may be valid for samples of people with ME/CFS in different parts of the world.

Our findings were built on earlier attempts to identify symptom-based clusters. For example, Hickie et al. showed that in CFS a distinction can be made between high and low symptomatic people [16]. Furthermore, using five common symptoms listed in the diagnostic criteria for CFS (i.e. muscle pain, joint pain, headaches, painful lymph nodes, sore throat) Collin et al. identified three phenotypes in a UK secondary care cohort (i.e. high symptomatic, low symptomatic and pain-only), which were replicated in another UK patient cohort and a Dutch tertiary care cohort [13, 27]. The existence of a highest symptomatic and lowest symptomatic clusters in ME/CFS was confirmed by our analyses.

Similar to Huber et al. we not only included the most commonly reported symptoms in ME/CFS, but we also included non-core symptoms [12]. In addition to the high and low symptomatic subgroups, they were able to identify symptom-specific subgroups, including one subgroup with primarily gastro-intestinal symptoms, one subgroup with primarily circulatory symptoms, one subgroup with gastro-intestinal and circulatory symptoms, and one subgroup with circulatory symptoms and orthostatic Intolerance [12].

In this study, symptoms were derived from a validated measure of ME/CFS symptomatology [19, 20], and both symptom frequency and severity scores were used in the analyses. Therefore, in contrast to earlier studies [12, 15, 18], our clusters were not only based on the presence of specific symptoms, but we were also able to identify subgroups of people with ME/CFS that were characterized by lower severity or frequency scores for specific symptoms.

This study used case definitions that are commonly used for the diagnosis of ME/CFS. The large majority of the patients (90%) fulfilled the Fukuda criteria, and there is a considerable overlap between the case definitions. Indeed, 29% of the patients met the criteria for all four different case definitions. However, there is a subset of patients that represent a symptom pattern which is only captured by specific case definitions. For example, 18 patients (5%) only fulfilled the ME-ICC case definition. Interestingly, another 5% of the participants did not meet the criteria of any of the four common case definitions. The lack of consensus in diagnostic criteria for ME/CFS underlines the difficulty in diagnosing the disease, and limits the external validity of ME/CFS studies using a specific case definition as inclusion criterion. In addition, different core symptoms are required for the different case definitions, which is also reflected in the identified symptom-based clusters. For example, none of the participants in Cluster 37 fulfilled the CCC or ME-ICC, explained by the fact that in these participants fatigue was not the result of exertion and they experienced no sensitivities, and a lack of neurosensory, flu-like, gastrointestinal and/or cardiovascular symptoms. In contrast, in Cluster 36 all participants met the ME-ICC, but only half of them met the Fukuda, CCC and IOM criteria, as half of the participants reported to experience lifelong fatigue [2].

It has been already been suggested that, instead of using the different ME/CFS case definitions, classification of patients according to severity and symptom patterns might be more useful to predict differences in prognosis or expected effects of therapy [28]. Additionally, identification of clusters of ME/CFS patients with distinct symptom patterns can provide more insight in the disease burden and may be useful for developing treatment strategies tailored to individual needs of patients.

### Strengths and limitations

A clear strength of this study is the use of two datasets with a considerable amount of participants with ME/CFS to identify and apply the symptom-based clusters. This supports the external validity of our findings. Furthermore, using the SOM approach, we were able to cluster a large dataset and visualize it on a two-dimensional map, in which similar datapoints are clustered into the same group or nearby groups.

This study has the following methodological limitations. First, the possibility of selection bias is present, as it is reasonable to assume that participants with a higher symptom burden are more likely to complete the questionnaire. On the other hand, patients with cognitive or concentration related problems are less likely to participate. To account for concentration related problems, participants were allowed to take breaks while completing the online questionnaire. Second, similar to earlier studies, the used populations predominantly consisted of females [29, 30], though it has been recognized that prevalence of ME/CFS is higher in women compared to men [31]. Furthermore, almost all participants were white, whilst however the prevalence of ME/CFS has suggested to be higher in ethnic minority populations [32, 33]. Third, the DSQ-2 only captures the frequency and severity of symptoms, but doesn’t account for within-day and between-day variation in symptoms. Future studies should consider the use of ecological momentary assessment, which involves repeated measurements of the participant’s symptoms, behavior and context in vivo and in real time [34]. Fourth, clusters were based on self-reported symptoms and did not include clinical variables, such as inflammatory markers, physical functioning, anxiety, depression, common comorbidities and/or current treatment. Moreover, the online survey did not allow us to identify whether symptom-based clusters were associated with relevant patient-reported outcomes, including functional status and quality of life.

## Conclusion

This study demonstrated that in ME/CFS there are subgroups of patients displaying a similar pattern of symptoms. These symptom-based clusters were confirmed in an independent ME/CFS sample. Classification of ME/CFS patients according to severity and symptoms patterns might be useful to develop tailored treatment options. Future studies are needed to investigate the relation between the identified symptom clusters and clinically relevant outcomes in patients with ME/CFS, including health-related quality of life and daily functioning.

## Supplementary Information

**Additional file 1.** Additional details on the clustering method.

**Additional file 2: Figure S1.** Number of reported symptoms in Dutch (n = 337) and US (n = 252) database using the 2/2 threshold for frequency and severity.

**Additional file 3: Table S1.** Symptom severity and frequency scores of the 13 largest clusters.

**Additional file 4: Table S2.** Symptom severity and frequency scores of the remaining 42 clusters with < 10 participants.

**Additional file 5: Table S3.** Participant characteristics of all clusters.

## Data Availability

The datasets used and/or analyzed during the current study are available from the corresponding author on reasonable request.

## Abbreviations

ME: Myalgic encephalomyelitis
CFS: Chronic fatigue syndrome
PEM: Post-exertional malaise
CCC: Canadian ME/CFS criteria
ME-ICC: ME International Consensus Criteria
IOM: Institute of Medicine
WMO: Medical Research Involving Human Subjects Act
METC azM/UM: Medical ethics committee of the University Hospital Maastricht and Maastricht University
DSQ-2: DePaul Symptom Questionnaire version 2
IQR: Interquartile ranges
SOM: Self-organizing map
