# Supplementary material for: Symptom-based clusters in people with ME/CFS: an illustration of clinical variety in a cross-sectional cohort
Source: J Transl Med. 2023 Feb 10;21:112. doi: 10.1186/s12967-023-03946-6 (PMC9921324; doi:10.1186/s12967-023-03946-6)
Supplement: Supplementary file 1 — Additional file 1. Additional details on the clustering method. [file 12967_2023_3946_MOESM1_ESM.docx]

**Additional material**

Symptom-based clusters in people with ME/CFS: an illustration of clinical variety

1. Self-Organizing Maps

Since each data point (information of one patient) has more than 150 features, including the frequency and severity of each COPD symptom, it is generally difficult to cluster patients into different groups due to two main challenges:

- It is impossible to visualize the distributions of data points with more than three features and, therefore, impossible to know the number of clusters/groups.
- It is difficult to interpret the clustering outcomes from the clinical perspective.

For clustering of high-dimensional data, there are four major types of algorithms, density-based, distribution-based, centroid-based, and hierarchical-based. Each clustering algorithm has its pros and cons, and the selection of algorithms depends on the structure of the data, training time, and accuracy. A comparison of the most commonly used clustering algorithm types can be found in Table 1.

Table 1 – Comparison of different types of clustering algorithms[1].

| **Algorithm Type** | **Description** | **Advantages** | **Disadvantages** |
| --- | --- | --- | --- |
| Density-Based | Group data points that are closely packed together | - Does not require to specify number of clusters beforehand - Robust to outliers - Can find arbitrarily shaped clusters. | - Sensitive to distance measure - Does not work well for data sets with large differences in densities |
| Distribution-Based | Group data points based on the probability they belong to a given cluster | - Simple and easy to implement - Easy to adjust for changes - Work well on categorical data | - A probability distribution needs to be known before clustering - Assume that all features are independent |
| Centroid-Based | Group data points based on multiple centroids in the data set | - Simple - Easy to adjust for changes - Efficient - Tight clusters | - Difficult to determine the optimal number of clusters - Not robust to outliers - Sensitive to the order of data - Limited to numerical data |
| Hierarchical-Based | Build a tree of clusters and organize the clusters from the top-down. | - The result is easy to interpret - Easy to implement | - Not efficient for a large data set - Sensitive to the order of the data - Very sensitive to outliers |

This study aims to cluster more than 330 COPD patients based on 158 features (frequency and severity of 79 symptoms) into different groups. Due to high dimensionality and significant differences in densities within the data set, density-based clustering algorithms do not work well since we either get a group of more than 300 patients or more than 300 groups with one patient. And similar issues happen for the hierarchical-based clustering algorithms. Distribution-based clustering algorithms are not suitable for the data set because we have no prior information about the distribution of data points. Therefore, we choose a centroid-based clustering algorithm – self-organizing maps (SOM). Besides, the reason for using SOM instead of other centroid-based algorithms to cluster COPD patients is that SOM can cluster high-dimensional data points and project them onto a low (usually two) dimensional map while preserving the topological structure of the data[2]. It means that similar data points will be clustered to the same group or nearby groups on the map, and eventually facilitates us studying the similarities among clusters.

A SOM is a type of artificial neural network (ANN). There are two major components (layers) in a SOM, an input layer to receive the input data and a two-dimensional ($m_{1}\times m_{2}$) competitive layer to determine which group a data point will be allocated. The competitive layer consists of $m_{1}\times m_{2}$ competitive units (neurons), and each competitive unit associates a weight (vector) that has the same dimension as the input data:

$W_{i}=\left\{ w_{i,1}, w_{i,2}, \ldots, w_{i,n} \right\}, i=1, 2, \ldots, m_{1}\times m_{2}$ ( 1 )

Each $W_{i}$ has to be initialized (with zero or any other random numbers) before training the SOM. Unlike other ANN types, a SOM does not have an activation function in neurons. Instead, it uses competitive learning to update its weight based on the following three processes:

- ***Competition****:* for each data point $A_{j}=\left\{ a_{j,1}, a_{j,2}, \ldots, a_{j,n} \right\}$, compute the minimum Euclidean distance between the data point and the competitive units (see Figure 1):

$\min_{W_{i}} d\left( W_{i}, A_{j} \right)=\min_{W_{i}} \left\| W_{i}-A_{j} \right\|=\min_{W_{i}} \left\{ \sqrt{\sum_{k=1}^{n} \left( w_{i,k}-a_{j,k} \right)^{2}} \right\}$ ( 2 )

We call the competitive unit $W_{i}$ with the shortest distance to the data point the best matching unit (BMU) of the competition at time $t$:

$W_{\mathrm{BMU}}=\underset{W_{i}}{\mathrm{argmin}} d\left( W_{i}, A_{j} \right)=\underset{W_{i}}{\mathrm{argmin}} \left\{ \sqrt{\sum_{k=1}^{n} \left( w_{i,k}-a_{j,k} \right)^{2}} \right\}$ ( 3 )

Figure 1: An illustration of finding the best matching unit (BMU) between a data point A_j_ and the competitive layer.

- ***Corporation****:* find the topological neighborhood of the BMU based on the following neighborhood kernel function (influence rate):

$\beta\left( W_{\mathrm{BMU}},W_{i} \right)=e^{-\frac{d\left( W_{\mathrm{BMU}},W_{i} \right)}{{2\sigma}^{2}\left( t \right)}}$ ( 4 )

And the neighborhood size decay rule:

$\sigma\left( t \right)=\sigma_{0}\left( t \right)e^{-\frac{t}{\lambda_{\sigma}}} , t=0, 1, 2, \ldots$ ( 5 )

Where:

$d\left( W_{\mathrm{BMU}},W_{i} \right)$– the lateral distance between BMU and the competitive unit $i$

$\sigma_{0}$– the radius of neighborhood at time zero ($t=0$)

$t$ – the current time step

$\lambda_{\sigma}$– the time constant

The neighborhood kernel function indicates that the influence of the learning decreases as the distance of a competitive unit from the BMU increases, and the topological neighborhood size decreases as time increases. As training goes on, the topological neighborhood gradually shrinks. At the end of the training, the neighborhood will shrink to zero size.

- ***Adaptation***: after finding the BMU among all competitive units and the associated topological neighborhood of the BMU, update the BMU and the competitive units within the topological neighborhood of the BMU:

$W_{i}\left( t+1 \right)=W_{i}\left( t \right)+\alpha\left( t \right)\beta\left( W_{\mathrm{BMU}},W_{i} \right)\left[ A_{j}-W_{i}\left( t \right) \right]$ ( 6 )

Where $\alpha\left( t \right)$ is the learning rate:

$\alpha\left( t \right)=\alpha_{0}\left( t \right)e^{-\frac{t}{\lambda_{\alpha}}} , t=0, 1, 2, \ldots$ ( 7 )

The output is a matrix of $m_{1}\times m_{2}$ rows and $n$ columns, where $m_{1}\times m_{2}$ indicates the number of competitive units and $n$ indicates the number of features. The detail of training an SOM can be found in Algorithm 1.

| **Initialization** | 1. Initialize all the competitive units, $t\leftarrow0$ and $j\leftarrow0$ |
| --- | --- |
| **Training** | 1. Repeat until convergence:    1. Select an input data point $A_{j}$    2. Find the best matching unit (BMU) for $A_{j}$ according to Eq. (1)    3. Find the topological neighborhood for $A_{j}$ according to Eq. (4) and Eq. (5)    4. Update the BMU of $A_{j}$ and the competitive units within the topological neighborhood of the BMU using Eq. (6) and (7)    5. $t\leftarrow t+1$ and $j\leftarrow j+1$ |

Algorithm 1: Self-Organizing Maps training algorithm[3].

1. Case Study

The clustering of COPD patients using SOM was performed on MATLAB, following its default setting except that we changed the number of iterations for training the SOM to 1000[3], and we used the default random number generation to initialize all competitive units.

- 1. Determine the Number of Clusters

Although there is no standard approach for finding the optimal number of competitive units, we can adapt the concept of identifying the number of cluster from other clustering algorithms such as K-Means. For example, one commonly used metric to determine the number of clusters in K-Means is the sum of squared errors (SSE):

$SSE=\sum_{k=1}^{K} \sum_{A_{l}\in C_{k}} \left\| A_{l}-m_{k} \right\|^{2}$

( 8 )

Where:

$C_{k}$– the $k^{\mathrm{th}}$ group in the SOM, $\forall_{i\neq j}C_{i}\cap C_{j}=\emptyset$, and $\bigcup_{k=1}^{K} C_{k}=X=\bigcup_{\forall l} \left\{ A_{l} \right\}$

$m_{k}$– the centroid of group $C_{k}$

$\left\| \cdot\right\|$ – Euclidean distance

However, using SSE to determine the number of competitive units has one drawback. SSE tends to zeros as the SOM dimensions ($m_{1}$ and $m_{2}$) increase. In the worst case, each group has at most one data point after clustering, the corresponding SSE will be 0. Therefore, we adapt the SSE to the following for SOM:

${SSE}_{c}=\frac{1}{N_{c}}\sum_{k=1}^{m_{1}\times m_{2}} \sum_{A_{l}\in C_{k}} \left\| A_{l}-m_{k} \right\|^{2}$

( 9 )

Where $N_{c}$ denotes the number of clusters that have more than two data points. ${SSE}_{c}$ can be interpreted as the average SSE per group (with more than two data points). This metric penalizes the situation of having large $m_{1}$ and $m_{2}$. It can be observed from Figure 2 that the optimal number of competitive units is 45 based on the lowest ${SSE}_{c}$, where $m_{1}=9$ and $m_{2}=5$.


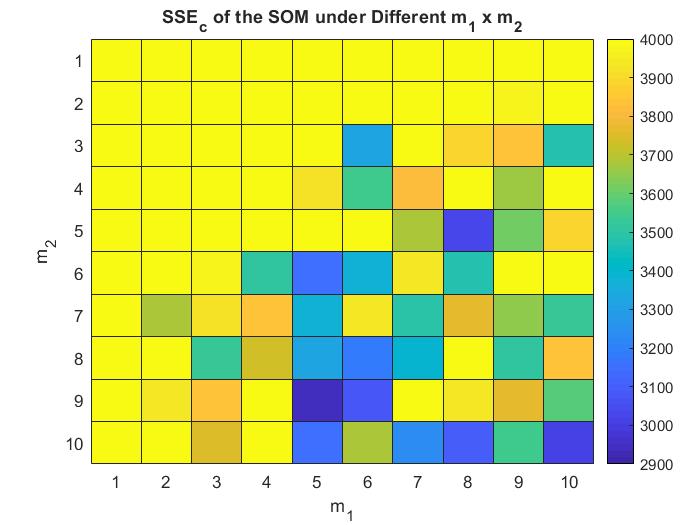


Figure 2: ${SSE}_{c}$ under different SOM dimensions.

- 1. Clustering Analysis

The previous subsection shows that the number of competitive units is 45 ($m_{1}=9$ and $m_{2}=5$), this gives us 13 groups (with more than two data points) among 45 clusters, as shown in Figure 3.


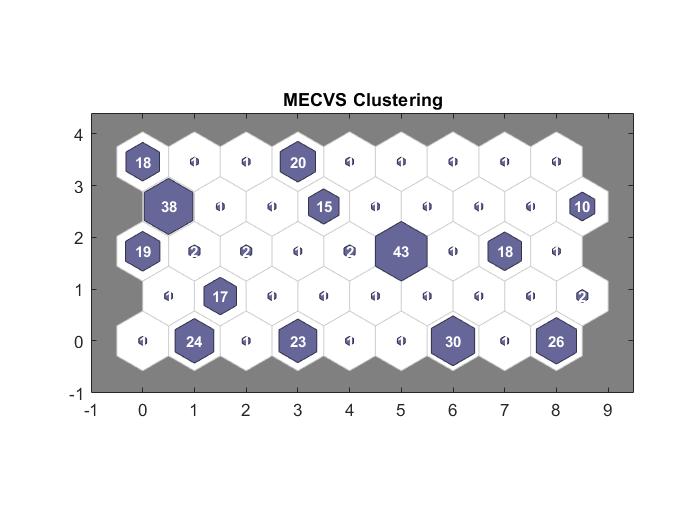


Figure 3: MECVS data clustering using self-organizing maps.

To evaluate the performance of the SOM on clustering MECVS data, we compare its results with K-Means algorithm (19 clusters) using the Davies-Bouldin index[4]:

|  | **SOM (45 Clusters)** | **K-Means (19 Clusters)** |
| --- | --- | --- |
| **Number of groups with more than two data points** | 13 | 13 |
| **Davies-Bouldin Index** | 3.3547 | 3.4781 |

Table 2: Comparison of clustering results from SOM and K-means using the Davies-Bouldin Index.

The Davies-Bouldin index is the average of the ratios between the within-cluster distance and the between-cluster distance of the worst-case scenario of all groups[4]. A lower Davies-Bouldin index indicates a better separation[4]. Thus, Table 2 shows that the SOM outperforms K-Means for clustering the MECVS data.

After clustering the MECVS data using the SOM approach, it is essential to find the top $n_{f}$ key features to describe each of the groups. This can be done by the following steps:

1. For each group (of more than two data points), the data points within the group are labelled as “1”, otherwise labeled as “0”.
2. Apply the Chi-Square feature selection method [5] to sort all the features in a descending order based on their chi-square values.
3. Select the top $n_{f}$ features (columns) from the output matrix of the SOM and use them to classify the data set.

Overall, the average classification accuracy for all groups (with more than two data points) increases if more top features are used, as shown in Figure 4. Since the most significant improvement occurs when the number of top features used in classification increases from four to five, we use the top five features of each group to define the group characteristics.


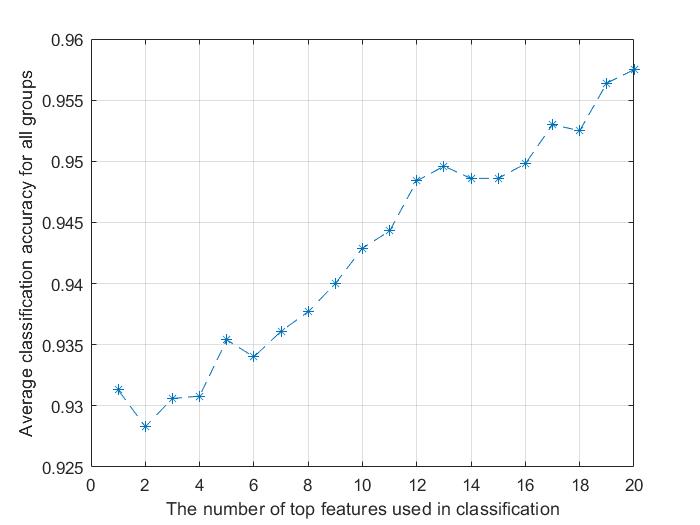


Figure 4: The relation between average classification accuracy and the number of top features used in classification.

**References**

1. McGregor M. 8 Clustering Algorithms in Machine Learning that All Data Scientists Should Know. freeCodeCamp2020.

2. Li J. Information Visualization with Self-Organizing Maps. SlideServe2019.

3. Bullinaria JA. Self Organizing Maps: Fundamentals. 2004.

4. Davies DL, Bouldin DW. A Cluster Separation Measure. IEEE Transactions on Pattern Analysis and Machine Intelligence 1979;PAMI-1(2):224-7.

5. Gupta A. Chi-Square Test for Feature Selection – Mathematical Explanation. GeeksforGeeks2019.
