# Supplementary material for: Symptom-based clusters in people with ME/CFS: an illustration of clinical variety in a cross-sectional cohort
Source: J Transl Med. 2023 Feb 10;21:112. doi: 10.1186/s12967-023-03946-6 (PMC9921324; doi:10.1186/s12967-023-03946-6)

**Figure S1** Number of reported symptoms in Dutch (n=337) and US (n=252) database using the 2/2 threshold for frequency and severity


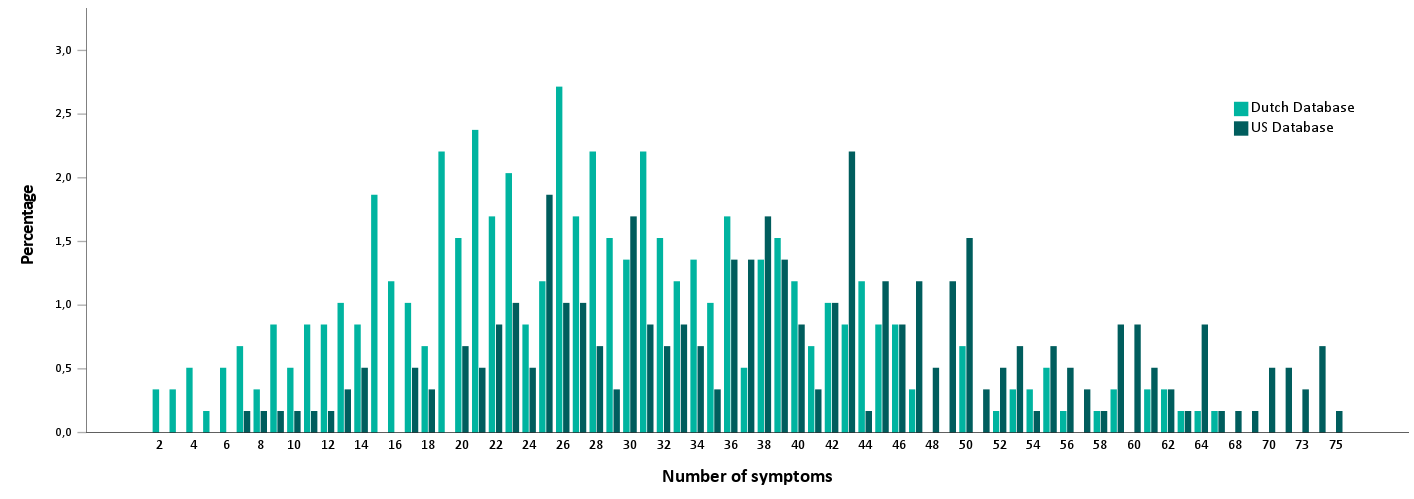

Supplement: Supplementary file 2 — Additional file 2: Figure S1. Number of reported symptoms in Dutch (n = 337) and US (n = 252) database using the 2/2 threshold for frequency and severity. [file 12967_2023_3946_MOESM2_ESM.docx]
